# A Versatile Plant Rhabdovirus-Based Vector for Gene Silencing, miRNA Expression and Depletion, and Antibody Production

**DOI:** 10.3389/fpls.2020.627880

**Published:** 2021-01-12

**Authors:** Xingxing Peng, Xiaonan Ma, Shuting Lu, Zhenghe Li

**Affiliations:** ^1^State Key Laboratory of Rice Biology, Institute of Biotechnology, Zhejiang University, Hangzhou, China; ^2^Ministry of Agriculture Key Laboratory of Molecular Biology of Crop Pathogens and Insect Pests, Zhejiang University, Hangzhou, China; ^3^Key Laboratory of Biology of Crop Pathogens and Insects of Zhejiang Province, Zhejiang University, Hangzhou, China

**Keywords:** viral vector, RNA silencing, VIGS, miRNA, antibody expression, plant rhabdovirus, sonchus yellow net virus

## Abstract

Plant virus vectors are ideal tools for delivery of genetic cargo into host cells for functional genomics studies and protein overexpression. Although a vast number of plant virus vectors have been developed for different purposes, the utility of a particular virus vector is generally limited. Here, we report a multipurpose plant rhabdovirus-based vector system suitable for a wide range of applications in *Nicotiana benthamiana*. We engineered sonchus yellow net rhabdovirus (SYNV)-based gene silencing vectors through expressing a sense, antisense, or double-stranded RNAs of target genes. Robust target gene silencing was also achieved with an SYNV vector expressing a designed artificial microRNA. In addition, ectopic expression of a short tandem target mimic RNA using the SYNV vector led to a significant depletion of the target miR165/166 and caused abnormal leaf development. More importantly, SYNV was able to harbor two expression cassettes that permitted simultaneous RNA silencing and overexpression of large reporter gene. This dual capacity vector also enabled systemic expression of a whole-molecule monoclonal antibody consisting of light and heavy chains. These results highlight the utility of the SYNV vector system in gene function studies and agricultural biotechnology and provide a technical template for developing similar vectors of other economically important plant rhabdoviruses.

## Introduction

In functional genomics studies, the interrogation of gene function is best achieved through genetic manipulation of gene expression and subsequently monitoring the resultant phenotypic changes. Common methodologies include generating targeted loss-of-function mutants, RNA silencing/RNA interference-based downregulation of gene expression, and transgenic (over)expression (Kuromori et al., 2009). Although currently available transgenic strategies have fulfilled many of these purposes in model plants, genetic manipulation of numerous recalcitrant crop species or varieties requires facile transformation-free approaches. Plant virus vectors provide ideal tools for transient delivery of genetic elements for alterations of plant gene expression in a manner largely independent of host genotypes. Owing to its simplicity, rapidity, and cost-effectiveness, transient plant viral vector approaches provide valuable alternatives to stable transformation and have become powerful technologies for gene function studies and biotechnological innovations (Palmer and Gleba, 2014; Cody and Scholthof, 2019; Dommes et al., 2019; Abrahamian et al., 2020).

One of the most significant technological avenues empowered by plant “vectorology” is virus-induced gene silencing (VIGS). By harnessing the natural RNA silencing antiviral defense response in plants, down-regulation of endogenous mRNAs is achieved at the post-transcriptional level through infection of host plants with a recombinant virus carrying a fragment of a target gene (Becker and Lange, 2010; Lacomme, 2014). Upon virus replication, RNA silencing is triggered by virally derived double-stranded RNA (dsRNA) synthesized by virus- and/or host-encoded RNA-dependent RNA polymerases. The dsRNAs are cleaved into 21∼24-nucleotide (nt) small interfering RNAs (siRNAs) by Dicer-like enzymes and then incorporated into Argonaute protein-containing RNA silencing-induced silencing complexes (RISCs) that target cognate viral and host mRNAs for degradation (Lacomme, 2014). During the past two decades, numerous VIGS vectors have been developed, and the ever-increasing list enables validation of gene function in a wide range of model and crop plant species (Huang et al., 2012; Lacomme, 2014; Dommes et al., 2019).

A technique analogous to VIGS is virus-mediated gene silencing through expression of natural microRNAs (miRNA) or artificial miRNAs (amiRNAs) (Chen et al., 2015). As with siRNAs, mature miRNAs of typically 21∼22 nt in length are incorporated into Argonaute proteins to direct target mRNA degradation. In plants, miRNAs are generated by Dicer-like 1 protein processing of 5’-capped, 3’-polyadenylated primary miRNA (pri-miRNA) precursors with imperfect self-complementary foldback structures (Bologna and Voinnet, 2014; Yu et al., 2017). The amiRNA technology utilizes the endogenous pri-miRNA backbones and exploits miRNA biogenesis pathway to produce artificially designed miRNAs that direct efficient and highly specific RNA silencing of the target genes of interest (Alvarez et al., 2006; Schwab et al., 2006). Because miRNA biogenesis involves nuclear processing of RNA polymerase II-produced pri-miRNA transcripts (Bologna and Voinnet, 2014; Yu et al., 2017), conventional approach to express miRNA/amiRNA employs a stably integrated transgene (Ossowski et al., 2008). However, the utility of plant virus miRNA/amiRNA vectors has also been demonstrated by using single-stranded DNA (ssDNA) geminiviruses that undergo replication and transcription in the nucleus (Tang et al., 2010; Gu et al., 2014; Ju et al., 2017). Notably, since many (but not all) geminivirus are phloem-limited, the loss-of-function phenotypes are more or less restricted to cells of the vascular system (Gu et al., 2014; Ju et al., 2017). Therefore, additional efficient miRNA viral vector systems with broad tissue tropism are needed.

While amiRNAs are ideal for gene knock-down, the functions of endogenous miRNAs can also be dissected genetically by loss-of-function approaches through transgenic expression of RNA decoys that compete for miRNA binding to target mRNAs. These non-coding RNAs, such as “target mimic” (Franco-Zorrilla et al., 2007), short tandem target mimic (STTM) (Yan et al., 2012), and “miRNA sponge” (Reichel et al., 2015), act to sequester or deplete miRNAs, thereby perturbing native miRNA-mediated target mRNA destruction. To avoid laborious and time-consuming transformation, several plant virus vectors have been exploited for transient expression of RNA decoys to block the activity of miRNAs belonging to diverse dicot and monocot families (Du et al., 2014; Gu et al., 2014; Sha et al., 2014; Yan et al., 2014; Jiao et al., 2015; Liao et al., 2015; Zhao et al., 2016; Jian et al., 2017).

Apart from their uses for the production of non-coding RNA (siRNAs, miRNAs/amiRNAs, and miRNA decoys), plant virus-based transient protein expression systems also provide a rapid tool for research on gene function and reprogramming of host physiology. Important applications include studies of cellular protein activity and localization (Cheuk and Houde, 2018), fast screening of host resistant proteins or pathogen effector proteins (Kanneganti et al., 2007; Lee et al., 2012), metabolic engineering (Zhang et al., 2013; Majer et al., 2017), and flowering timing regulation (Lin et al., 2007; McGarry et al., 2017). Additionally, due to the extraordinary speed, yield, and scalability, transient virus expression systems hold great promise for manufacturing massive amounts of high-value proteins/polypeptides, e.g., vaccine antigens or immunoglobulins (Ig), for veterinary and medical uses (Gleba et al., 2007; Marsian and Lomonossoff, 2016; Abrahamian et al., 2020). Despite these merits, currently developed infectious plant virus vectors generally tolerate relatively small inserts and are unable to stably express large proteins (Gleba et al., 2007; Peyret and Lomonossoff, 2015). Therefore, development of novel vector systems with greater cargo capacity and genome plasticity promises to unleash the greater potentials for gene function studies and biotechnology applications.

Sonchus yellow net virus (SYNV) is a non-segmented negative-stranded RNA virus (NSV) belonging to the genus *Nucleorhabdovirus*, family *Rhabdoviridae*. The genome of SYNV contains six open reading frames (ORFs) in the order 3’-N-P-sc4-M-G-L-5’ that are conserved among plant rhabdoviruses (Jackson et al., 2005). The N, P, L proteins form a ribonucleoprotein complex with the viral genomic RNA that is essential for replication and transcription (Ganesan et al., 2013; Wang et al., 2015), and the M and G proteins are involved in virus budding and morphogenesis (Sun et al., 2018), whereas the sc4 protein is the cell-to-cell movement protein (Zhou et al., 2019). Several genetic features of rhabdovirus make their genomes highly amenable for vector engineering. First, the genome is organized in a linear, modular form. The generally non-overlapping ORFs are flanked by short non-coding leader (le) and trailer (tr) regions at the 3’- and 5’- ends of the genome that provide cis-acting elements for viral RNA synthesis and genome encapsidation. Located between each ORF are clearly defined gene-junction sequences that signal transcription termination of the upstream mRNAs and reinitiation of downstream mRNA transcription (Jackson et al., 2005). Additional foreign expression cassettes can be readily engineered into the viral genome under the control of the transcriptional cis-elements. Second, viral mRNA transcription occurs sequentially from the 3’ end of the genomic RNA and attenuates progressively after each successive downstream gene junction to produce discrete monocistronic mRNA species in a decreasing gradient, i.e., N > P > M > G > L (Whelan et al., 2004; Jackson et al., 2005). The polar transcriptional mechanism permits regulated levels of foreign gene expression by adjusting the gene insertion site in the viral genome (Tokusumi et al., 2002; Wertz et al., 2002). Each viral mRNA is structurally indistinguishable at the 5’ and 3’ termini from cellular mRNAs in that they are capped and polyadenylated during transcription.

NSVs generally display greater genetic stability and cargo capacity than most positive-stranded RNA viruses (PSVs) (Finke and Conzelmann, 2005; Bukreyev et al., 2006). Several animal rhabdoviruses have been widely used as vectors for vaccine development, gene therapy, and cancer virotherapy. With recent developments in reverse genetics, recombinant engineering of plant rhabdoviruses has become possible, and previous reports have shown that reporter genes can be inserted at multiple genomic locations and stably expressed (Wang et al., 2015; Qian et al., 2017; Gao et al., 2019; Ma et al., 2020). In this study, we report engineering of a multipurpose SYNV vector for expression of various non-coding RNAs and production of heterologous proteins. The utility of these SYNV vectors are demonstrated by their capacities for expression and assembly of heterooligomeric proteins, or simultaneous RNA silencing and protein overexpression. Our study combines the repertoire of available viral vector applications into a single streamlined virus vector set.

## Materials and Methods

### Plant Growth and Agroinfiltration

*N. benthamiana* plants were grown in a growth chamber at 25°C under a 16 h light/8 h dark cycle. Agrobacteria EHA105 cultures containing the SYNV infectious clones were grown overnight in Luria-Bertani (LB) media and sedimented by centrifuging. The pellet was resuspended in MES buffer (10 mm MgCl_2_, 10 mm MES, pH 5.6, 150 μM acetosyringone) and adjusted to the concentrations of 0.7 density at OD600. To recover recombinant SYNV vectors, equal volumes of the agrobacterial suspensions carrying the pGD-NPL plasmid for expression of the N, P, and L core proteins (Wang et al., 2015), the pCB301-2b-p19-HcPro-γb plasmid for expression of viral suppressor of RNA silencing (Sun et al., 2017), and full-length SYNV infectious clone derivatives were mixed at OD600 of 0.7 and infiltrated into *N. benthamiana* leaves.

### Plasmids Constructions

The SYNV-GFP plasmid containing the GFP cassette has been described previously (Wang et al., 2015; Ma and Li, 2020) and was used as the founding clone to engineer SYNV vectors with various inserts between the *N* and *P* genes described in this study. There are two *Nco*I sites flanking the *GFP* gene to facilitate sequence replacement with the In-Fusion cloning method (Clontech, Japan). All primer sequences for cloning are listed in Supplementary Table S1.

To generate the SYNV-sGFP plasmid, we amplified a 409-bp m*GFP5* 3’ coding sequence by PCR from total DNA sample of *N. benthamiana* 16c plants using the primers NPJ-sGFP/F and sGFP-NPJ/R. The fragment was inserted into the *Nco*I-digested SYNV-GFP vector by using an In-Fusion cloning kit (Clontech, Japan). To generate the SYNV-asGFP plasmid, we amplified an inverted asGFP fragment with an identical sequence to the sGFP using primers NPJ-asGFP/F and asGFP-NPJ/R and then inserted the fragment into the SYNV-GFP vector to replace the *GFP* gene. To generate the SYNV-hpGFP plasmid, the sGFP fragment, a 131-bp intron sequence of Arabidopsis At1g05760 gene, and the asGFP fragment were amplified with the primer pairs NPJ-sGFP/F and sGFP-intron/R, intron/F and intron/R, and intron-asGFP/F and asGFP-NPJ/R, respectively. The three fragments were inserted into the linearized SYNV-GFP vector by In-fusion cloning. For the *PDS* gene silencing vectors, we chose the sequence from nucleotide coordinates no. 774 to 1182 of the *PDS* coding region to amplify the sense, antisense, and inverted repeats of the *PDS* sequences. The sPDS, asPDS, and hpPDS fragments were inserted into the SYNV-GFP vector as described above to generate SYNV-sPDS, SYNV-asPDS, and SYNV-hpPDS, respectively.

To engineer the SYNV-amiRPDS plasmid, we conducted sequential PCR reactions to generate a chimeric amiRNA sequence containing the backbone sequence of the Arabidopsis miR319a precursor gene (At4g23713) with the mature miRNA sequence substituted for a 21-nt sequence targeting the *PDS* gene (5#-UCAACAUAGACUGAUUGGGGC-3#). A detailed protocol for designing the amiRPDS fragment can be found in Tang et al. (2010). Briefly, three partially overlapping products were obtained in a first round of PCR reactions by using the primer pairs oligo A and PDS-IV, PDS-III and PDS-II, or PDS-I and Oligo B, respectively. The three PCR products were isolated, mixed, annealed and extended in a second round of PCR reactions, and the full-length fragment was amplified by using the Oligo A and B flanking primers. The final product was cloned into *Nco*I-digested SYNV-GFP by In-Fusion cloning. To generate the SYNV-STTM165/166 plasmid, the STTM165/166 sequence was amplified from the pCLCrV-STTM plasmid (Gu et al., 2014) using the primers STTM165/F and STTM165/R, and then the products were inserted into the *Nco*I-digested SYNV-GFP by In-Fusion cloning.

To generate the dual capacity plasmids, we amplified the GFP-RFP expression cassette from the SYNV-MR-GFP-RFP plasmid (Wang et al., 2015) with primers NPJ-GFP/F and RFP-NPJ/R, and recovered the GUS and hpGFP cassettes from pCambia1301 and SYNV-hpGFP with the primers pairs NPJ-GUS/F and GUS/R, and GUS-NPJ/F and asGFP-NPJ/R, respectively. These fragments were separately cloned into the *Nco*I-digested SYNV-GFP plasmid by In-Fusion cloning to generate the SYNV-GFP-RFP and SYNV-GUS-hpGFP plasmids, respectively.

For construction of SYNV vectors for expression of mAb, the light chain (LC) and heavy chain (HC) genes of the monoclonal IgG clone #8 specific for CMV coat protein (Zein et al., 2010) were chemically synthesized by GenScript (Nanjing, China). To facilitate expression in *N. benthamiana*, the LC and HC genes (GenBank accession nos. EF672201 and EF672215) were tobacco codon optimized and fused to the endoplasmic reticulum-targeting C-terminal SEKEDL sequence (Supplementary Table S2). The optimized LC and HC genes were cloned into the *Nco*I-digested SYNV-GFP plasmid by In-fusion cloning to produce the SYNV-LC_*N/P*_ and SYNV-HC_*N/P*_ intermediate plasmids. To generate SYNV-LC-HC_*N/P*_, we amplified the fragment spanning the LC coding region and the N/P gene junction sequence from the SYNV-LC_*N/P*_ plasmid using the primers NPJ-LC/F and LC/R, and the HC coding sequence from the SYNV-HC_*N/P*_ plasmid with the primers LC-NPJ/F and HC-NPJ/R, respectively. The two fragments were cloned into the *Nco*I-digested SYNV-GFP vector by In-Fusion cloning. Similar strategies were used to construct the SYNV-HC-LC_*N/P*_ plasmid.

To generate the SYNV-LC-HC_*le/N*_ plasmid, we utilized two unique restriction sites, the *Pvu*I in the leader sequence and the *Bsu*36I in the *N* coding region, in the SYNV-GFP_*le/N*_ cDNA clone (Qian et al., 2017) to facilitate subcloning. Fragment I containing the region through the *Pvu*I site to the 5’ untranslated region of the *N* gene and Fragment III spanning the N/P gene junction and the *Bsu*36I site were amplified by PCR from the SYNV-GFP_*le/N*_ plasmid using the primer pairs *Pvu*I/F with N5’UTR/R and NPJ/F and N-*Bsu*36I/R, respectively. Meanwhile, Fragment II containing the LC, N/P gene junction, and HC coding region was amplified from SYNV-LC-HC_*N/P*_ plasmid using primers N5’UTR-LC/F and HC-NPJ/R. The three fragments were inserted into the SYNV-GFP_*le/N*_ plasmid that were double-digested by *Pvu*I and *Bsu*36I by using In-Fusion cloning. The SYNV-HC-LC_*le/N*_ was constructed by the same strategy as used for SYNV-LC-HC_*le/N*_, with the exception that Fragment II containing the HC, N/P gene junction, and LC coding region was amplified from SYNV-HC-LC_*N/P*_ plasmid using the primers N5’UTR-HC/F and LC-NPJ/R.

### Quantitative Real-Time Reverse Transcription-PCR (qRT-PCR) and End-Point Stem-Loop RT-PCR

Total RNAs were extracted from SYNV-infected *N. benthamiana* leaves with Trizol reagent (Invitrogen, Grand Island, NY, United States). First-strand cDNAs were synthesized from these RNAs with a reverse transcription kit (Promega, Madison, WI). qRT-PCR reactions were performed in a LightCycler 480 real-time PCR instrument (Roche, Rotkreuz, Switzerland) and SYBR Green I Master kit (Roche, Rotkreuz, Switzerland). The miRNAs were detected via an end-point stem-loop RT-PCR method (Varkonyi-Gasic, 2017).

### GFP Imaging and Fluorescence Microscopy

Leaves or whole plants expressing GFP were illuminated under a hand-carried UV B-100AP lamp (UVP, Upland, CA) and photographed with a Nikon D80 digital camera. Fluorescence microscopy was performed with a Zeiss SteREO Lumar. V12 epifluorescence microscope using the filter sets Lumar 38 (excitation 470/40 nm; emission 525/50) for GFP and Lumar 31 (excitation 565/30 nm; emission 620/60 nm) for RFP. The images were processed with LSM software Zen 2009 (Carl Zeiss, Germany).

### GUS Staining

GUS expression was measured by staining for enzymatic activity with a β*-galactosidase* reporter gene staining Kit (Solarbio Life Sciences, Beijing, China). GUS staining was carried out at 37°C for a maximum of 12 hours, and the chlorophyll of green tissue was removed by repeated incubation in 70% ethanol at 37°C until satisfactory results were obtained.

### Protein Extraction and Western Blotting

Samples (100 mg) of 16c and *N. benthamiana* leaves were ground in liquid nitrogen with 200 μl of protein extraction buffer (0.1M Tris, 9M Urea, 4.5% SDS [wt/vol] and 7.5% β-mercaptoethanol [vol/vol], pH 6.8). Proteins separated by SDS-PAGE were either stained with Coomassie Brilliant Blue or transferred to nitrocellulose membranes (GE Healthcare, Piscataway, NJ) and probed with the antibodies against GFP or mouse IgG (whole-molecule) (Abcam, Cambridge, United Kingdom).

### Purification of Plant-Derived Recombinant mAbs

Samples (20 g) of *N. benthamiana* were ground in liquid nitrogen with 50 ml of protein extraction buffer (0.1M Tris, 150 mM NaCl, and 0.1% Tween 20 [vol/vol], pH 8.0) and the soluble proteins were extracted. The mAbs from the crude protein extracts of SYNV-LC-HC_*N/P*_ infected *N. benthamiana* leaf tissues were recovered by using a one-step affinity purification with Protein G magnetic beads (New England Biolabs, Beverly, MA) according to the instructions of the manufacturer. The concentrations of the purified mAbs preparations were quantified by Bradford assay.

### ELISA and mAb Quantification

Ninety-six-well Immuno Plates (Thermo Fisher, Waltham, United States) were coated with CMV-infected *N. glutinosa* leaves extracted in sodium carbonate buffer (15 mM Na_2_CO_3_, 35 mM NaHCO_3_, pH 10.0). The wells were blocked with 250 μl of PBST buffer (137 mM NaCl, 2.7 mM KCl, 10 mM Na_2_HPO_4_, 1.8 mM KH_2_PO_4_, 0.2% Tween-20 [vol/vol], pH 7.2) containing 5% skim milk powder (wt/vol). SYNV-infected leaf tissue (100 mg) was homogenized in 200 μl of protein-extraction buffer (0.1 M Tris-Cl, 150 mM NaCl, and 0.1% Tween-20 [vol/vol], pH 8.0). A mouse hybridoma-derived CMV-specific mAb (Yu et al., 2005) was used as a positive control at a dilution of 1:10,000 in PBST buffer. Plates were loaded with 100 μl of crude plant extracts or diluted mAb solution, incubated at 37°C for 1 hour, and washed three times with PBST buffer. After incubation with alkaline phosphatase-conjugated goat anti-mouse IgG (whole-molecule) antibodies (Cat. # ab6708, Abcam, Cambridge, United Kingdom), plates were treated with 100 μl of developing solution containing 4-Nitrophenyl phosphate disodium salt hexahydrate (Sigma-Aldrich, Saint Louis, MO, United States) as a substrate. The color reaction was quenched with 100 μl of 3 M NaOH. Absorbance was measured at 405 nm by using a universal microplate reader EL800 (Bio-Tek Instruments, Winooski, VT). To quantify the levels of mAbs expressed in infected *N. benthamiana* tissues by ELISA, serially diluted samples of the above purified mAb were loaded in triplicates to ELISA plates alongside with the total protein extracts to be measured. Once the intensity of each well had been recorded on the plate reader, we calculated the average absorbance values for each triplicate. A standard curve was generated based on the absorbance values for the purified mAb samples, which were used to determine the concentrations of the assembled full-size mAbs expressed in the leaf extracts.

## Results

### Construction of SYNV VIGS Vectors and Silencing of GFP Transgene Expression in *N. benthamiana*

**FIGURE 1:**
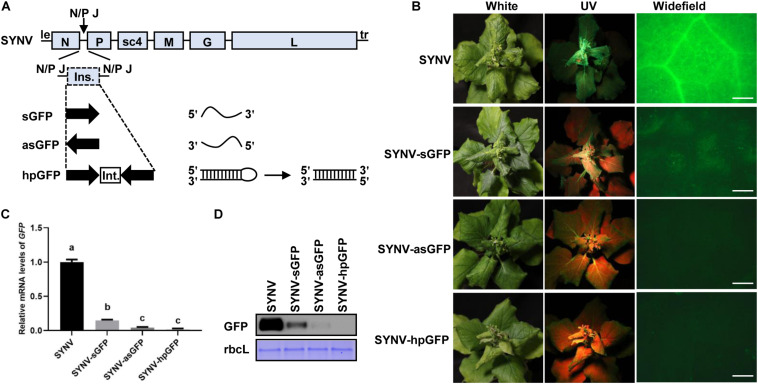
Silencing of the *GFP* transgene in *Nicotiana benthamiana* 16c plants by SYNV VIGS vectors. **(A)** Schematic diagram of the genome organization of sonchus yellow net virus (SYNV) and strategies for construction of VIGS vectors. le, leader; tr, trailer; N/P J, N/P gene junction; ins., insert; int., intron; sGFP, sense GFP; asGFP, antisense GFP; hpGFP, hairpin GFP. **(B)** Observation of green fluorescence in plants systemically infect with SYNV VIGS vectors at 14 days after systemic infection (dpsi). Infected plants were photographed under white light (left panels) or long-wavelength UV light (middle panels). GFP fluorescence in epidermal cells of upper emerging leaves was also captured by an epifluorescence microscopy (right panels). Scale bar = 2 mm. **(C)** qRT PCR analysis of GFP mRNA levels in systemically infected leaf tissues at 14 dpsi. Data are means of three infected plants (*n* = 3). Error bars show the standard deviation (± SD). Different letters represent significant differences at *p* < 0.05 according to one-way ANOVA followed by Tukey’s multiple comparisons test. **(D)** Western blot analysis of GFP expression in systemically infected leaf tissue at 14 dpsi. The large RuBisCO subunit (rbcL) was stained by Coomassie Brilliant Blue to indicate equal protein loading.

Silencing of plant gene expression can be efficiently triggered by infection of host plants with viral vectors expressing sense, antisense, or inverted repeated RNA sequences of host target genes (Huang et al., 2012; Lacomme, 2014). To determine whether the SYNV vector could induce gene silencing and to investigate the efficiency of different types of silencing RNA, we designed SYNV vectors to target a *green fluorescent protein* (*GFP*) transgene in *N. benthamiana* 16c plants (Ruiz et al., 1998). A 409-base pair (bp) fragment corresponding to a portion of the *GFP* coding region was inserted into the infectious SYNV cDNA clone in either the sense or antisense orientation to generate SYNV-sGFP and SYNV-asGFP. In addition, a viral construct (SYNV-hpGFP) containing the sense and antisense *GFP* fragments flanking a plant intron sequence was also engineered. Each of these fragments was inserted into the SYNV genome as an additional transcription unit (ATU) between the *N* and *P* genes by positioning them downstream of a duplicated N/P gene junction sequence to ensure that capped, polyadenylated RNA species derived from the inserts were transcribed during virus replication (Figure 1A). Notably, because SYNV and other nucleorhabdoviruses undergo genome replication and mRNA transcription in the infected nuclei (Jackson et al., 2005), the intron sequence in the primary hpGFP transcripts were anticipated to be spliced out by host nuclear pre-mRNA splicing machinery to generate intermolecularly base-paired dsRNAs (Figure 1A).

*N. benthamiana* 16c plants were inoculated with the individual SYNV vectors via agroinfiltration. At approximately 15 days after agroinoculation, typical SYNV symptoms were observed in agroinfiltrated plants, confirming successful virus infections. At 14 days post systemic infection (dpsi), GFP expression was monitored by UV illumination of the infected plants and by epifluorescence microscopy of newly expanded leaves (Figure 1B). Expression of GFP appeared to be efficiently suppressed in SYNV-sGFP-infected leaf tissues, albeit faint GFP fluorescence was still visible. In contrast, infections with either SYNV-asGFP or SYNV-hpGFP resulted in robust silencing, leading to nearly undetectable GFP expression by fluorescence imaging. To more precisely determine the silencing efficiency, we measured the *GFP* mRNA and protein levels in GFP-silenced leaves by qRT-PCR and Western blotting. Compared to wild-type SYNV infections, the *GFP* mRNA levels in leaf tissues infected with SYNV-sGFP, SYNV-asGFP, and SYNV-hpGFP were decreased to 15%, 4%, and 2%, respectively (Figure 1C). These data are consistent with the GFP imaging experiments and are further supported by protein gel blot analysis (Figure 1D). In conclusion, these results show that *GFP* transgene expression is silenced most efficiently by negative-strand SYNV vectors expressing a hairpin or antisense RNA, and less efficiently by a vector expressing sense RNA.

### Silencing of the *N. benthamiana* Endogenous *Phytoene Desaturase* (*PDS*) Gene

Previous studies have shown that transgenes are more susceptible to VIGS than endogenous genes (Ruiz et al., 1998; Vaistij et al., 2002). To evaluate the ability of the SYNV-derived vectors to knock-down endogenous gene expression, we next targeted the *N. benthamiana PDS* gene, whose silencing provides a convenient chlorophyll photo-bleaching phenotype for visual inspection (Kumagai et al., 1995; Ratcliff et al., 2001). As with the above-described SYNV-based GFP silencing constructs, a 409-bp fragment correspondingly to a portion of the *PDS* coding region was inserted into the SYNV clones to produce VIGS vectors carrying sense (SYNV-sPDS), antisense (SYNV-asPDS), or intron-containing inverted-repeats of the *PDS* sequence (SYNV-hpPDS).

**FIGURE 2:**
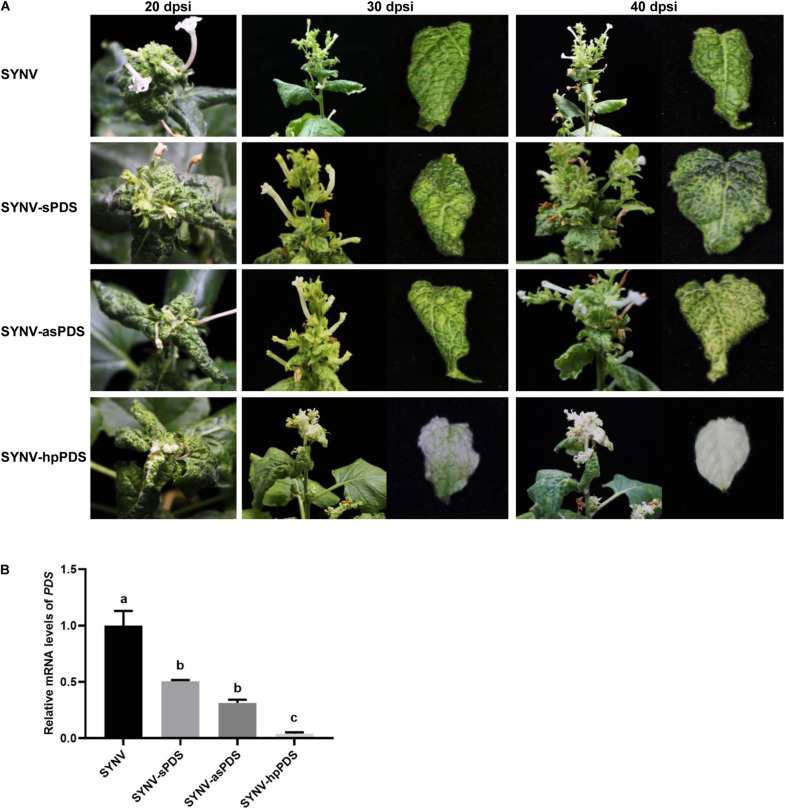
Silencing of *N. benthamiana PDS* gene by SYNV VIGS vectors. **(A)** Symptoms and silencing phenotypes of plants infected by SYNV vector expressing a sense (SYNV-sPDS), antisense (SYNV-asPDS), or hairpin RNA (SYNV-hpPDS) of a portion of the *PDS* gene at 20, 30 and 40 dpsi. **(B)** qRT-PCR analysis of the relative mRNA levels of *PDS* in systemically infected leaf tissues at 40 dpi. Data are the means of three independently infected plants (*n* = 3). Error bars show the standard deviation (± SD). Different letters represent significant differences at *p* < 0.05 according to one-way ANOVA followed by Tukey’s multiple comparisons test.

After agroinfiltration of the viral vectors into the lower leaves of *N. benthamiana* plants, the phenotypes of infected plants were monitored in time-course experiments. As shown in Figure 2A, at 20 dpsi, discernable photo-bleaching started to appear in newly emerging leaves of SYNV-hpPDS infected plants but was absent in plants infected by SYNV-sPDS, SYNV-asPDS, or the SYNV control. As SYNV-hpPDS infections proceeded, extensive photo-bleaching spread to most mesophyll tissues in the young expanding leaves at 30 dpsi and eventually to the entire leaves by 40 dpsi (Figure 2A). In contrast, SYNV-asPDS infections only resulted in leaf chlorosis in the young leaves by 40 dpi, and similar but even weaker phenotypes were observed in plants infected with SYNV-sPDS. The chlorosis phenotype was prominent in young leaf tissues adjacent to the major and minor veins. However, these leaves failed to develop the typical photo-bleaching phenotype up to the time of leaf senesce. A qRT-PCR assay was conducted to measure the amounts of *PDS* mRNA in the leaves with the most pronounced silencing phenotype at 40 dpsi. Consistent with the observed phenotypes, the expression of *PDS* was reduced to 3% by infections with SYNV-hpPDS, 34% with SYNV-asPDS, and 58% with SYNV-sPDS, in comparison to SYNV control samples (Figure 2B). Thus, these data show that SYNV vectors expressing either sense or antisense RNA trigger silencing of endogenous gene expression relatively inefficiently, but that the efficiency was greatly enhanced after transcription of a hairpin RNA.

### Silencing of *PDS* Gene With an SYNV amiRNA Vector

**FIGURE 3:**
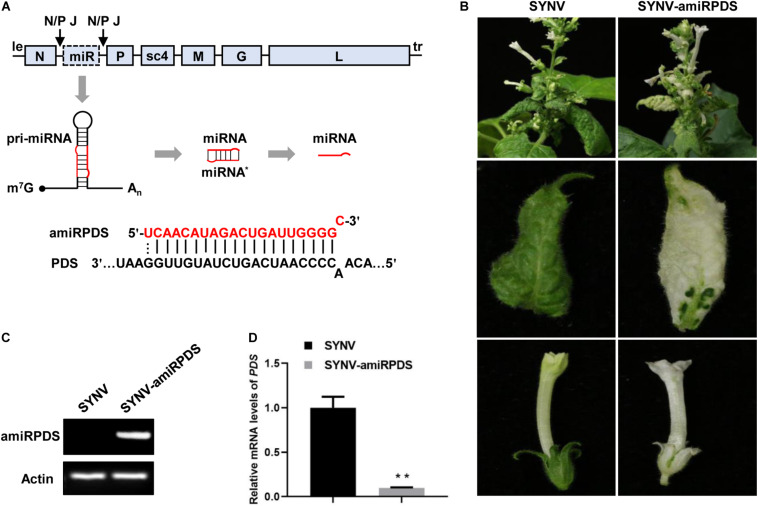
*PDS* gene silencing in *N. benthamiana* by SYNV amiRNA vector. **(A)** Schematic representation of amiRNA expression and the base pairing between amiRPDS and the target PDS mRNA. m^7^G: cap; A_*n*_: poly(A) tails. miR, microRNA. **(B)** Phenotypes of plants infected with SYNV and SYNV-amiRPDS at 30 dpsi. **(C)** End-point stem-loop RT-PCR analysis of mature amiRPDS levels in systemically infected leaf tissue at 30 dpsi. The *actin* gene was used as an internal control. **(D)** qRT-PCR analysis of *PDS* mRNA levels in upper leaf tissues of infected plants at 30 dpsi. Data are the means of three infected plants (*n* = 3). Error bars show the standard deviation (± SD). Double asterisk (**) indicate significant difference at *p* < 0.01 according to Student’s *t*-test.

Nucleorhabdoviruses, such as SYNV, replicate and transcribe 5’ capped and 3’ polyadenylated mRNAs in the nucleus (Jackson et al., 2005), which parallels the RNA structures and subcellular compartment of pre-miRNA transcription. Therefore, we reasoned that the SYNV-based vector would be suitable for expression of miRNA/amiRNA to induce specific silencing of target genes. To this end, we constructed an SYNV vector carrying an engineered ATU between the *N* and *P* genes to express an amiRNA. We used the *Arabidopsis* miRNA319a precursor sequence as the amiRNA backbone. Within the precursor, the mature miRNA sequence was replaced by a 21-nt sequence targeting *PDS* mRNA (amiRPDS) (Figure 3A) because this amiRPDS species has been shown to silence *N. benthamiana PDS* expression specifically and efficiently (Tang et al., 2010). Upon SYNV replication and mRNA transcription, the pri-amiRPDS transcripts were anticipated to be processed to mature amiRPDS by the canonical miRNA maturation pathway (Figure 3A).

By 30 dpsi, *N. benthamiana* plants infected with the SYNV-amiRPDS vector developed an obvious photo-bleaching phenotype in the upper young leaves and flowers (Figure 3B). The extent of photo-bleaching induced by SYNV-amiRPDS was similar to that of SYNV-hpPDS, but was much greater than photobleaching elicited by SYNV-asPDS or SYNV-sPDS at the same time point (30dpsi) (compare Figure 3B with Figure 2A, middle panels). An end-point stem-loop RT-PCR assay conducted at 30 dpsi verified successful expression of amiRPDS in plants infected with SYNV-amiRPDS (Figure 3C), and qRT-PCR analysis confirmed a concomitant 90% reduction of *PDS* mRNA levels compared to the SYNV control samples (Figure 3D). In conclusion, our results show that SYNV-mediated expression of a single amiRNAs species induced gene silencing as robust as the SYNV hpRNA-containing VIGS vector in *N. benthamiana*.

### Depletion of miR165/166 With an SYNV STTM Vector

**FIGURE 4:**
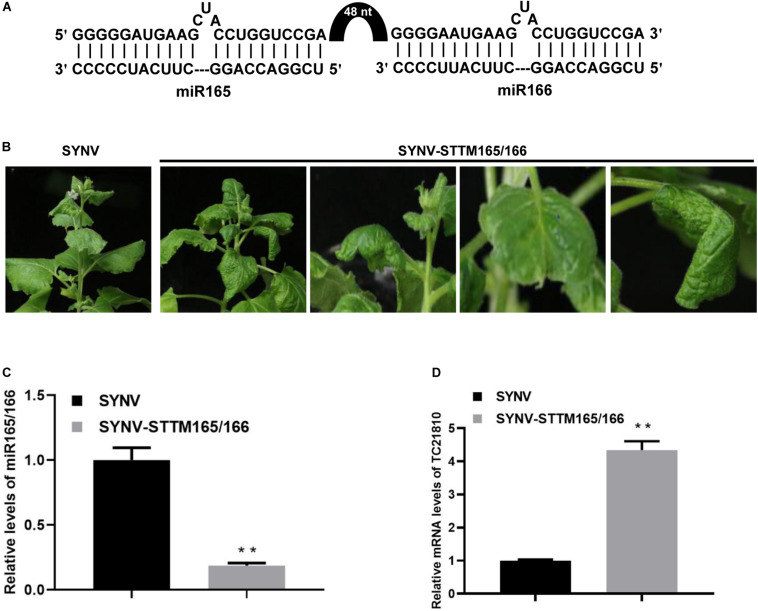
Suppression of miR165/166 by SYNV-STTM165/166 in *N. benthamiana.* **(A)** Schematic representation of base pairing between the STTM165/166 and miR165/166. The black arch represents the 48-nt stem-loop linker. **(B)** Phenotypes of plants infected with SYNV-STTM165/166 at 10 dpsi. Note: oval-shape and splitting along the edges of the blades of young emerging leaves, and downward bending of the midribs toward the stem. **(C,D)** Relative accumulation of miR165/166 **(C)** and miR165/166 target gene TC21810 **(D)** in systemically infected leaf tissue at 10 dpsi. Data are the means of three infected plants (*n* = 3). Error bars represent the standard deviation (± SD). Double asterisk (**) indicate significant difference at *p* < 0.01 according to Student’s *t*-test.

Having shown the utility of SYNV in amiRNA expression, we next tested SYNV vector-based silencing of endogenous miRNAs in *N. benthamiana* by use of the STTM technology (Yan et al., 2012). We targeted *N. benthamiana* miR165/166, an evolutionarily conserved miRNA family involved in regulation of leaf adaxial–abaxial patterning through repression of Class III homeodomain-leucine zipper transcription factors (Kidner and Martienssen, 2004; Mallory et al., 2004; Sakaguchi and Watanabe, 2012). An STTM sequence containing two miR165 and miR166 binding sites flanking a 48-nt partial stem-loop spacer was inserted into the SYNV vector as an ATU to produce the SYNV-STTM165/166 vector (Figure 4A) via a strategy similar to that described in Figure 1A. At 10 dpsi, *N. benthamiana* plants agroinoculated with SYNV-STTM165/166 exhibited abnormal leaf development, as evidenced by the oval-shaped upper developing leaves. Occasionally some leaves were observed to split at their edges or the midribs bent toward the stem (Figure 4B). These phenotypes are consistent with perturbed leaf polarity determination resulting from miR165/166 depletion. Notably, we did not observe ectopic outgrowths or enations on the midribs as previously shown when the STTM165/166 sequence was expressed from PSV vectors (Sha et al., 2014; Liao et al., 2015; Zhao et al., 2016). This discrepancy may be due to differential STTM expression levels and/or the combinatorial effects of virus-induced symptoms. Nevertheless, the qRT-PCR analyses revealed a 5.2-fold reduction in miR165/166 level in SYNV-STTM165/166 infected plants compared to SYNV-infected plants (Figure 4C). Accordingly, the depletion of miR165/166 was associated with a 4.3-fold increase in mRNA levels of the predicted target *TC21810* homeodomain-leucine zipper gene (Figure 4D).

### Simultaneous Protein Overexpression and Gene Silencing

**FIGURE 5:**
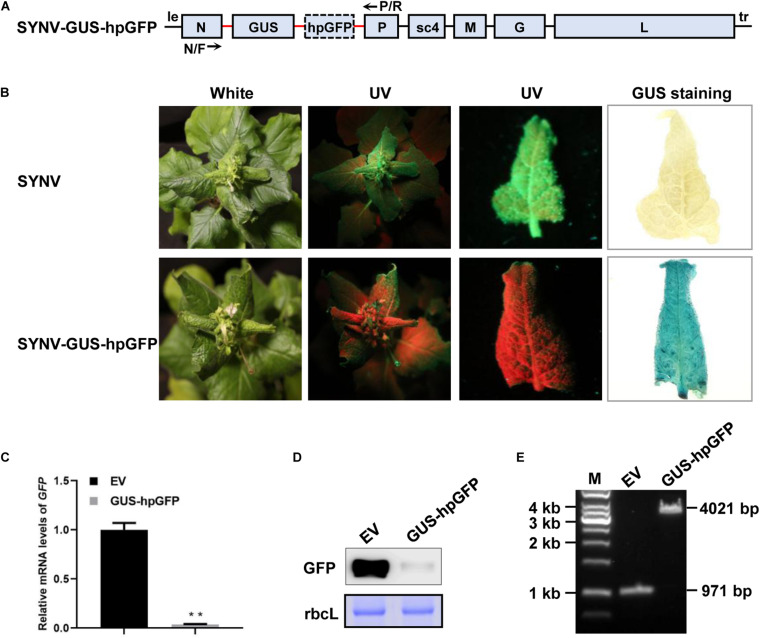
Simultaneous *GUS* gene expression and *GFP* silencing. **(A)** Schematic illustration of the SYNV genome organization and the strategy of GUS and hpGFP expression. The native and duplicated N/P gene junction sequences (N/P J) are depicted by the red line. The positions of the N/F and P/R primers used for RT-PCR detection of insert integrity are indicated. **(B)** Visualization of GFP fluorescence and GUS staining in systemically infected leaf tissues at 7 dpsi. The infected plants were photographed under white light and long-wavelength UV illumination. Upper systemically infected leaves were photographed under UV light and stained for GUS expression. **(C)** qRT-PCR assay to determine relative *GFP* mRNA levels in systemic leaves of plants infected with SYNV (EV) and SYNV-GUS-hpGFP (GUS-hpGFP) at 7 dpsi. Data are the means of three infected plants (*n* = 3). Error bars show the standard deviation (± SD). Double asterisk (**) indicate significant difference at *p* < 0.01 according to Student’s *t*-test. **(D)** Western blot analysis of GFP protein levels in systemically infected leaf tissues at 7 dpsi. The large RuBisCO subunit (rbcL) was stained with Coomassie Brilliant Blue to indicate equal protein loading. **(E)** RT-PCR analysis of insert stability. Total RNA was isolated from the upper infected leaves shown in (B) and was subjected to RT-PCR analysis with the N/F and P/R primers. A 1-kb DNA ladder marker is shown on the left and calculated sizes of PCR fragments are indicated on the right.

In certain scenarios during functional genomics studies, it is desirable to knock-down one gene while overexpressing another. To test whether SYNV can be used for simultaneous protein overexpression and gene silencing, we inserted a β*-glucuronidase (GUS)* gene expression cassette under the control of an additionally duplicated N/P gene junction into the SYNV-hpGFP silencing vector to produce SYNV-GUS-hpGFP (Figure 5A). Upon agroinfection of *N. benthamiana* 16c plants, SYNV-GUS-hpGFP induced robust systemic GFP silencing combined with high levels of GUS expression in the upper non-inoculated leaves at 7 dpsi, as revealed by GFP imaging and GUS staining (Figure 5B). The qRT-PCR and Western blot analyses of extracts from these leaves indicated that SYNV-GUS-hpGFP infection resulted in a 94% reduction in GFP mRNA level (Figures 5C,D), which was comparable to the silencing efficiency induced by SYNV-hpGFP infections (Figure 1C). RT-PCR assay with primers annealing to the flanking *N* and *P* genes yielded a single DNA fragment of approximately ∼ 4-kilobases (kb), indicating that SYNV-GUS-hpGFP stably maintained the intact RNA insert (3050 nts) during replication in the systemically infected plant tissue (Figure 5E). Therefore, the simultaneous expression of an additional gene does not appear to compromise the ability of SYNV-GUS-hpGFP to trigger potent RNA silencing.

### Expression of a Full-Size Monoclonal Antibody

**FIGURE 6:**
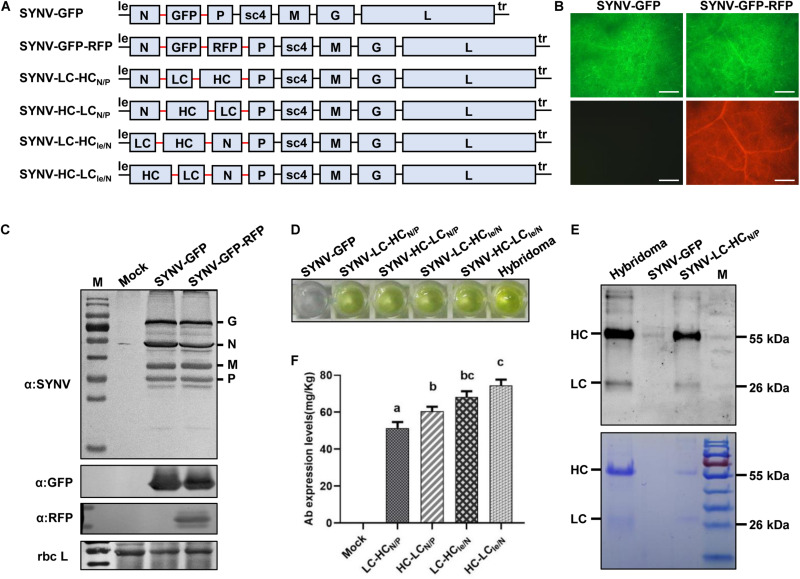
Engineering infectious SYNV vectors for expression of monoclonal antibody (mAb). **(A)** Schematic representation of SYNV-based dual expression vectors. LC, light chain; HC, heavy chain. The N/P gene junction sequences (N/P J) are depicted by the red line. **(B)** Visualization of fluorescent reporter protein expression in systemic leaves of plants infected with SYNV-GFP and SYNV-GFP-RFP at 10 dpsi. Scale bar = 500 μm. **(C)** Protein gel blots showing the expression of SYNV structural proteins and fluorescent reporter proteins in the leaves shown in **(B)**. **(D)** ELISA detection of cucumber mosaic virus-specific mAb in SYNV-infected leaf tissue. The hybridoma-derived mAb serves as a positive control. **(E)** Analysis of mAbs purified from the SYNV-LC-HC_*N/P*_ leaf extract by protein gel blotting (upper panel) and Coomassie Brilliant Blue staining (lower panel). The positions of HC and LC polypeptides are indicated along with a protein size marker (M). **(F)** Quantification of mAb titers in *N. benthamiana* leaf tissues infected with SYNV vectors harboring HC and LC gene insertions. The concentrations (mg/kg leaf fresh weight) of the assembled CMV-specific mAb were determined by ELISA and quantified with a standard curve. Mock represents values in leaf extracts of SYNV-GFP infected plants. Data are the means of three individually infected plants (*n* = 3). Error bars show the standard deviation (± SD). Different letters represent significant differences at *p* < 0.05 according to one-way ANOVA followed by Tukey’s multiple comparisons test.

We have previously engineered an SYNV-GFP vector capable of stable expression of a *GFP* reporter gene during multiple plant-to-plant transfers (Wang et al., 2015). To test whether SYNV-based vectors could be used to co-express two proteins simultaneously, a second ATU encoding a *red fluorescent protein* (RFP) was inserted into SYNV-GFP to generate the SYNV-GFP-RFP vector. Both GFP and RFP were expressed under the control of duplicated N/P gene junctions and inserted between the viral *N* and *P* genes in the SYNV genome (Figure 6A). Fluorescent microscopy and Western blot analyses confirmed high GFP and RFP expression levels throughout the emerging leaves of SYNV-GFP-RFP infected plants (Figures 6B,C), and the expression of the reporter genes was stable after repeated mechanical transmissions. Insertion of the second ATU did not appear to affect the viability or titer of the virus vector, since the levels of viral structural proteins and the GFP protein were similar in SYNV-GFP and SYNV-GFP-RFP protein extracts (Figure 6C).

The construction of the dual-reporter SYNV vector enabled us to test the expression and assembly of heterooligomeric proteins or protein complexes in the same infected cells. We chose to express a full-size monoclonal Immunoglobulin G (IgG) protein that consists of two identical LC and HC polypeptides. For this purpose, the GFP and RFP coding sequences in SYNV-GFP-RFP were replaced with the LC and HC genes, respectively, of a monoclonal antibody (mAb) against cucumber mosaic virus (CMV) to generate the SYNV-LC-HC_*N/P*_ vector (Figure 6A). After agroinoculation of *N. benthamiana* with SYNV-LC-HC_*N/P*_ constructs, crude protein extracts were isolated from the upper systemically infected leaves to determine the expression and assembly of the functional IgG molecules. An antigen-specific Enzyme-Linked Immunosorbent Assay (ELISA) assay revealed that SYNV-LC-HC_*N/P*_ protein extracts reacted specifically with CMV antigen (Figure 6D), indicating successful expression and assembly of the functional CMV mAb. As a negative control, protein extracts prepared from SYNV-GFP-infected plants displayed minimal background reaction. Saps of SYNV-LC-HC_*N/P*_ plants were used to infect healthy *N. benthamiana* through mechanical inoculation to obtain large batch of infected plants. Whole mAb molecules were purified from the infected leaf tissues by Protein G affinity chromatography. Separation of the purified mAbs in denaturing SDS-PAGE gels, followed by protein gel blotting and Coomassie Brilliant Blue staining, revealed that the HC and LC subunits derived from plants were equivalent in size to a CMV-specific mAb obtained from mouse hybridoma cells (Figure 6E).

According to the rhabdovirus mRNA polar transcription mechanism, more abundant mRNAs are made from the leader (le)-proximal genes, and transcription is attenuated progressively toward the downstream trailer (tr)-proximal genes (Whelan et al., 2004; Qian et al., 2017). In an attempt to improve IgG production and assembly by altering the HC and LC expression levels, we constructed another viral vector SYNV-HC-LC_*N/P*_ that differs from SYNV-LC-HC_*N/P*_ in the order of LC and HC genes. Additionally, the LC and HC cassettes in SYNV-LC-HC_*N/P*_ and SYNV-HC-LC_*N/P*_ were relocated to the genomic loci between the leader and *N* gene to produce the SYNV-LC-HC_*le/N*_ and SYNV-HC-LC_*le/N*_ vectors, respectively (Figure 6A). These viral constructs were individually delivered into *N. benthamiana* leaves through agroinfiltration, and recombinant viruses were successfully recovered in infiltrated plants with similar efficiencies and elicited symptoms typical of SYNV infections. We confirmed the expression of CMV-specific mAbs in systemic leaves of plants infected with the four SYNV vectors by ELISA (Figure 6D). The concentrations of fully-assembled mAb in leaf extracts were measured by ELISA and quantified by using an established standard curve. The yields of CMV-specific mAb in leaf extracts of SYNV-LC-HC_*N/P*_, SYNV-HC-LC_*N/P*_, SYNV-LC-HC_*le/N*_, and SYNV-HC-LC_*le/N*_ were determined to be 51.4, 60.6, 68.2, and 74.4 mg per kg of fresh weight, respectively (Figure 6F). Thus, viral vectors with mAbs genes positioned between the leader and *N* gene (SYNV-LC-HC_*le/N*_ and SYNV-HC-LC_*le/N*_) yielded assembled IgG approximately 23–33% higher than those inserted between the *N* and *P* genes (SYNV-LC-HC_*N/P*_ and SYNV-HC-LC_*N/P*_). In comparison, constructs in which the HC precedes the LC (resulting in more abundant HC than LC mRNAs) appeared to be slightly more efficient in producing functional mAbs than those with the reverse order (Figure 6F). In conclusion, SYNV-based-vectors with two engineered ATUs are able to express heterooligomeric proteins, and higher expression levels were achieved with SYNV vectors in which ATUs were inserted at the 3’ proximal end of the genome.

## Discussion

Members of plant-adapted rhabdoviruses collectively infect diverse dicotyledonous and monocotyledonous crops, as well as economically important woody plant species such as citrus, apple, peach, persimmon, papaya, raspberry, and coffee (Jackson et al., 2005; Dietzgen et al., 2020). Facile genetic tools for analyses of most of these hosts are lacking, so development of infectious rhabdovirus vector systems will provide valuable solutions for functional genomics studies. We and others have shown that SYNV and barley yellow striate mosaic virus (BYSMV) are promising rhabdovirus vector candidates for development of functional genomic tools (Wang et al., 2015; Gao et al., 2019; Ma et al., 2020). In this study, we have extended recombinant rhabdovirus engineering to develop a versatile SYNV vector system for RNA silencing, miRNA expression and depletion, and foreign protein expression. We have used an “add-a-gene” strategy to insert exogenous genetic cargos into the SYNV genome as ATUs driven by a duplicated gene junction sequence. The results also show that two engineered ATUs can be inserted in tandem between the le/N or the N/P intergenic regions to enable simultaneous protein expression and VIGS, or expression and assembly of heterodimeric IgG proteins. For some animal-infecting non-segmented NSVs, foreign expression cassettes can also be inserted into downstream genomic loci, such as those between the P/M, M/G, G/L, or L/trailer, to achieve progressively lower amounts of expression as a consequence of polar mRNA transcription (Tokusumi et al., 2002; Wertz et al., 2002; Zhao and Peeters, 2003). Although not being investigated in the present study, it is possible that similar SYNV genomic locations will also be amenable to foreign gene insertion to permit fine-tuned expression levels of foreign proteins. Therefore, SYNV versatility and engineering flexibility provides an attractive vector candidate for validation of endogenous gene/miRNA functions.

Another advantage of the SYNV-based vectors over most other plant virus vectors is their remarkable genetic stability and large cargo capacity. We previously reported that the engineered GFP cassette in the recombinant SYNV-GFP remains stable over multiple rounds of serial mechanical transmission (Wang et al., 2015). For the vectors constructed in this study, we also observed no sign of insert loss, despite cargoes consisting of one or two duplicated gene junction sequences and inserts of up to 3 kbs. The unrivaled cargo capacity and genome stability of SYNV is further demonstrated by our recent study showing that foreign CRISPR/Cas9 inserts of approximately 5 kbs are stably maintained in progeny virions after mechanical passages (Ma et al., 2020). These characteristics are in direct contrast to most PSV vectors, which generally accommodate relatively small inserts and frequently eliminate foreign genes during systemic infections or after plant-to-plant transfers (Scholthof et al., 1996; Gleba et al., 2007). These insert instability issues are particularly relevant when large foreign sequences are inserted and when a homologous subgenomic RNA promoter is employed to drive gene expression (Beck and Dawson, 1990; Donson et al., 1991; Avesani et al., 2007; Chung et al., 2007). The introduction of a homologous sequence into PSV genomes facilitates recombination during genome replication through template-switching mechanisms (Nagy and Simon, 1997), which leads to purging of foreign sequence. However, unlike the naked genomes of PSVs, NSV genomes are encapsidated to form nucleocapsids during replication. During viral RNA synthesis, only a short stretch of nucleotides within the nucleocapsids are unveiled and recognized by viral polymerase for genome replication and mRNA transcription (Ivanov et al., 2011; Ruigrok et al., 2011; Jackson and Li, 2016). Consequently, recombination of NSVs are rare and genome instability is minimized (Chare et al., 2003; Han and Worobey, 2011). Besides, the helical architecture of rhabdovirus nucleocapsids provides substantial elongation flexibility to accommodate large cargoes (Schnell et al., 1996; Ma et al., 2020). Thus, rhabdovirus virion architecture and replication strategies provide unparalleled insert stability and cargo capacity for vector development.

VIGS represents one of the most widespread applications of virus-based genomics tools, and currently, many VIGS systems derived from PSVs and DNA viruses are available for a wide range of plant species. In this study, we have shown that SYNV can be engineered to elicit efficient silencing of endogenous host gene and transgene in *N. benthamiana*, thus expanding the VIGS vector portfolio to include a plant NSV. Investigation of the requirements of SYNV VIGS vectors in initiation of potent host gene silencing revealed that expression of hairpin RNAs is required, suggesting that dsRNA formation is a limiting step in the SYNV VIGS system. The antisense triggers, albeit less efficient than the hairpin constructs, are more efficient than the sense triggers. These data may be explained by base-pairing of antisense RNAs with cellular mRNAs to increase the pool of dsRNA triggers. However, for most PSV and DNA virus vectors, either sense or antisense triggers are generally sufficient to induce robust RNA silencing, although the silencing efficiencies in some cases can be further enhanced through the expression of inverted-repeats (Lacomme et al., 2003; Hein et al., 2005; Pflieger et al., 2008). It is commonly presumed that abundant dsRNAs are produced as replication intermediates during PSV genome replication, whereas ssDNA virus infections generate 3’ end overlapping transcripts due to convergent bidirectional transcription from circular viral genomes. Because NSV genome encapsidation is concomitant with viral replication, it is conceivable that dsRNA formation is minimal. Indeed, abundant dsRNAs were detected in cells infected with PSVs or DNA viruses, whereas no or only weak dsRNA signals were observed during NSV infections (Weber et al., 2006; Son et al., 2015).

Given the requirement of hairpin RNA construction for robust RNA silencing and the protracted disease onset (∼ 2 weeks), the utility of the SYNV-based VIGS vectors in the model *N. benthamiana* plants is unlikely to match other well-established VIGS systems such as tobacco rattle virus and potato virus X (Baulcombe, 1999; Bachan and Dinesh-Kumar, 2012). Also, SYNV has a rather limited host range and induces obvious symptoms, which constrain its applications as a VIGS vector. However, the large cargo capacity of SYNV permits simultaneous RNA silencing and expression of large foreign genes. This dual capacity is desirable in functional genomics studies in which two-way alterations of gene expression are needed.

Another utility of the SYNV-based vectors is to express amiRNA, which directed specific gene silencing as robust as the hairpin RNA VIGS vector that produced a ∼409-bp dsRNA. Long dsRNA precursors can generate an extensive collection of siRNA species, some of which may adventitiously silence unintended mRNAs and result in off-target effects (Jackson and Linsley, 2004; Senthil-Kumar and Mysore, 2011). By contrast, amiRNA technology utilized only a single 21-nt small RNA species whose actions can be more readily predicted than those of diverse siRNAs generated from long dsRNAs (Ossowski et al., 2008). Moreover, although dsRNA-mediated RNA silencing may target any homologous gene, expression of a tailored miRNA can discriminate between highly similar alleles or spliced forms, or between closely related members in a gene family (Ossowski et al., 2008). Despite these advantages, viral vector-based transient expression of amiRNA has previously been limited to nuclear ssDNA geminiviruses, many of which have restrictive phloem tropisms. Our experiments have extended this technology to an NSV rhabdoviruses that invade mesophyll tissues to result in more widespread silencing phenotypes. We anticipate that the SYNV-based amiRNA technology can be transferred to other nucleorhabdoviruses and dichorhabdoviruses that replicate in the nuclei of diverse crop species.

The ability of SYNV vectors to express multiple proteins will also permit easy and fast screening of protein complex functions *in vivo* and manufacturing of multi-subunit antibodies and vaccine antigens. As a proof-of-concept for these applications, we have shown that HC and LC genes inserted into various SYNV genomic loci are stably expressed and assembled into full-sized IgG molecules. The majority of current plant viral vectors generally accommodate only one expression cassette with limited lengths, although some exceptions have been described (Dolja and Koonin, 2013; Cheuk and Houde, 2018; Choi et al., 2019; Gao et al., 2019; Jiang et al., 2019). Simultaneous expression of two proteins with separate fully infectious virus vectors faces complications of virus-virus interactions that may affect protein production, e.g., antagonism, superinfection exclusion, and disparate tissue tropism and infection dynamics (Syller, 2012). Although these issues can be alleviated by the use of non-competing deconstructed viral replicon-based vectors, they are incapable of systemic movement and are thus unable to deliver the cargos to distal plant tissues (Gleba et al., 2014). In comparison, fully infectious rhabdovirus vectors such as SYNV have merits of systemic expression of target genes and easy inoculation of large numbers of plants using initially infected leaf tissues as launching pads. Despite of these advantages, the yields of assembled mAb obtained with the SYNV vector system is several fold lower than those by using the PSV replicon based vectors (about 200–500 mg/kg fresh weight) (Giritch et al., 2006). It remains to be tested whether fusion of a translational enhancer, such as tobacco mosaic virus omega leader sequence (Gallie et al., 1987), can improve the mAb expression by the SYNV vectors.

## Conclusion

In conclusion, the SYNV vectors developed here, together with the recently described derivatives for DNA-free delivery of CRISPR/Cas9 (Ma et al., 2020), provide novel and versatile combinations of applications in plant functional genomics and biotechnology. Our studies also pave the way for development of similar vector systems with other plant rhabdoviruses that infect economically important crop plants or fruit trees. In addition, the majority of plant rhabdoviruses are transmitted in a circulative, propagative manner by sap-sucking planthopper, leafhopper, and aphids (Whitfield et al., 2018). Therefore, developments in rhabdovirus vectors also hold promise for molecular biology studies of these otherwise genetically intractable agricultural pests.

## Data Availability Statement

The original contributions presented in the study are included in the article/Supplementary Material, further inquiries can be directed to the corresponding author/s.

## Author Contributions

ZL and XP conceived the project and designed the experiments. XP, XM, and SL performed the experiments. ZL and XP analyzed the data and interpreted the results. ZL and XP wrote the manuscript. All authors contributed to the article and approved the submitted version.

## Conflict of Interest

The authors declare that the research was conducted in the absence of any commercial or financial relationships that could be construed as a potential conflict of interest.
