# Supplementary material for: A Versatile Plant Rhabdovirus-Based Vector for Gene Silencing, miRNA Expression and Depletion, and Antibody Production
Source: Front Plant Sci. 2021 Jan 12;11:627880. doi: 10.3389/fpls.2020.627880 (PMC7835261; doi:10.3389/fpls.2020.627880)
Supplement: Supplementary file 1 [file Table_1.DOCX]

Supplementary Material

**Supplementary Table S1. Primers used in this study**

| Primer Name | Primer sequence (5'→3') | Description |
| --- | --- | --- |
| NPJ-GFP/F | tatttgtctaggccATGGTGAGCAAGGGCGAG | Construction of SYNV-GFP-RFP |
| RFP-NPJ/R | ctgtagtttaccatgTTACAGGAACAGGTGGTGGC |  |
| NPJ-LC/F | tatttgtctaggccATGGGATGGTCTTGTATCATCC | Construction of SYNV-LC_N/P_ |
| LC-NPJ/R | ctgtagtttaccatgTCACAATTCATCCTTCTCAGAGCAC |  |
| NPJ-HC/F | tatttgtctaggccATGGGTTGGTCATGTATCATCC | Construction of SYNV-HC_N/P_ |
| HC-NPJ/R | ctgtagtttaccatgTCACAACTCATCTTTTTCAGACTTTC |  |
| LC/R | TCACAATTCATCCTTCTCAGAGCAC | Construction of SYNV-LC-HC_N/P_ |
| LC-NPJ/F | aaggatgaattgtgaTAAACTACAGCCACAACTCTAC |  |
| HC/R | TCACAACTCATCTTTTTCAGACTTTC | Construction of SYNV-HC-LC_N/P_ |
| HC-NPJ/F | aaagatgagttgtgaTAAACTACAGCCACAACTCTAC |  |
| PvuI/F | TGCCAAATGTTTGAACGATCG | Construction of SYNV-LC-HC_le/N_ and SYNV-HC-LC_le/N_ |
| N5’UTR/R | GATTACCTGCAATTAAAATACTCAAAAAATACG |  |
| NPJ/F | TAAACTACAGCCACAACTCTAC |  |
| N-Bsu36I/R | CTGAAAGAACAAGAACCTGAGGAC |  |
| N5’UTR-LC/F | taattgcaggtaatcATGGGATGGTCTTGTATCATCC | Construction of SYNV-LC-HC_le/N_ |
| N5’UTR-HC/F | taattgcaggtaatcATGGGTTGGTCATGTATCATCC | Construction of SYNV-HC-LC_le/N_ |
| NPJ-sGFP/F | tttgtctaggccatgGACACGTGCTGAAGTCAAG | Construction of SYNV-sGFP |
| sGFP-NPJ/R | ctgtagtttaccatgTTAAAGCTCATCATGTTTGTATAG |  |
| NPJ-asGFP/F | tttgtctaggccatgTTAAAGCTCATCATGTTTGTATAG | Construction of SYNV-asGFP |
| asGFP-NPJ/R | ctgtagtttaccatgGACACGTGCTGAAGTCAAG |  |
| sGFP-intron/R | tcagacttacaacgtTTAAAGCTCATCATGTTTGTATAG | Construction of SYNV-hpGFP and SYNV-hpPDS |
| intron-asGFP/F | cccagcagatagagcTTAAAGCTCATCATGTTTGTATAG |  |
| intron/F | ACGTTGTAAGTCTGATTTTTGACTC |  |
| intron/R | GCTCTATCTGCTGGGTCC |  |
| NPJ-sPDS/F | tttgtctaggccatgGGCACTTAACTTCATAAACCCTG | Construction of SYNV-sPDS |
| sPDS-NPJ/R | ctgtagtttaccatgCTTCAGTTTTCTGTCAAACCATATATG |  |
| NPJ-asPDS/F | tttgtctaggccatgCTTCAGTTTTCTGTCAAACCATATATG | Construction of SYNV-asPDS |
| asPDS-NPJ/R | ctgtagtttaccatgGGCACTTAACTTCATAAACCCTG |  |
| sPDS-intron/R | tcagacttacaacgtCTTCAGTTTTCTGTCAAACCATATATG | Construction of SYNV-hpPDS |
| intron-asPDS/F | cccagcagatagagcCTTCAGTTTTCTGTCAAACCATATAT |  |
| GFP/qRT/F | GGTGAAGGTGATGCAACATACGG | qRT-PCR analysis of GFP expression |
| GFP/qRT/R | TGAAGAAGATGGTCCTCTCCTGC |  |
| Oligo A | CTGCAAGGCGATTAAGTTGGGTAAC | Construction of SYNV-amiRPDS |
| Oligo B | GCGGATAACAATTTCACACAGGAAACAG |  |
| NPJ-amiRPDS/F | tttgtctaggccatgCAAACACACGCTCGGACGC |  |
| amiRPDS-NPJ/R | ctgtagtttaccatgCATGGCGATGCCTTAAATAAAGAT |  |
| PDS-I | GATCAACATAGACTGATTGGGGCTCTCTCTTTTGTATTCC |  |
| PDS-II | GAGCCCCAATCAGTCTATGTTGATCAAAGAGAATCAATGA |  |
| PDS-III | GAGCACCAATCAGTCAATGTTGTTCACAGGTCGTGATATG |  |
| PDS-IV | GAACAACATTGACTGATTGGTGCTCTACATATATATTCCT |  |
| NPJ-GUS/F | tttgtctaggccatgGTAGATCTGAGGGTAAATTTCTAG | Construction of SYNV-GUS-hpGFP |
| GUS/R | TCAGCTAGCTTGTTTGCCTC |  |
| GUS-NPJ/F | aaacaagctagctgaTAAACTACAGCCACAACTCTAC |  |
| STTM165/F | tttgtctaggccatgGGGGGATGAAGCTACCTGGTCCGAGTTGTTGTTGTTATGG | Construction of SYNV- STTM165/166 |
| STTM166/R | ctgtagtttaccatgTCGGACCAGGTAGCTTCATTCCCCATTCTTCTTCTTTAGACCA |  |
| PDS/qRT/F | ACACATCTGATAATCTGCTCTTCAGC | qRT-PCR analysis of PDS expression |
| PDS/qRT/R | CCGACAGGGTTCACAACCTG |  |
| amiRPDS/RT | GTTGGCTCTGGTGCAGGGTCCGAGGTATTCGCACCAGAGCCAACGCCCCA | RT-PCR of amiRPDS |
| amiRPDS/F | CCGGCTCAACATAGACTGAT | qRT-PCR analysis of miRPDS |
| Universal/R | GTGCAGGGTCCGAGGT | qRT-PCR analysis of miRPDS and miR165/166 expression |
| miR165/166/RT | GTCGTATCCAGTGCAGGGTCCGAGGTATTCGCACTGGATACGACGGGGAA | RT-PCR analysis of miR165/166 |
| miR165/166 /F | TCGGACCAGGCTTCATYCCCC | qRT-PCR analysis of miR165/166 expression |
| TC21810/F | ATCCGCCAAGGGATGCTAGT | qRT-PCR analysis of TC21810 expression |
| TC21810/R | TCGGCTCTAGACCAACCAGG |  |
| actin/F | CAATCCAGACACCTGTACTTTCTCTC | qRT-PCR analysis of Actin expression |
| actin/R | AAGCTGCAGGTATCCATGAGACTA |  |

Lowercase letters represent the homologous sequence to facilitate In-Fusion cloning.

**Supplementary Table S2. Sequences of the light chain and heavy chain genes of the CMV-specific mAb.**

| Light chain  (5'→3') | **ATG**GGATGGTCTTGTATCATCCTTTTTCTTGTTGCTACTGCTACAGGTGTTCATTCAGAGCTTGATGTTGTTATGACTCAAACACCACTTACATTGTCTGTTACTATTGGACAACCTGCTTCTATTTCATGCAAGTCTTCACAATCACTTTTGGATTCTGATGGAAAAACTTATCTTAACTGGCTTTTGCAAAGACCAGGTCAATCACCTAAGAGGCTTATATATCTTGTTTCTAAGCTTGATTCTGGTGTTCCAGATAGGTTTACTGGTTCTGGATCAGGTACAGATTTTACTCTTAAGATTTCTAGGGTTGAAGCTGAGGATTTGGGAGTTTATTACTGTTGGCAAGGTACACATTTTCCATATACTTTTGGAGGTGGAACAAAGCTTGAGATTAAAAGAGCTGATGCTGCTCCTACTGTTTCAATTTTTCCACCTTCTTCAGAACAACTTACATCAGGTGGAGCTTCTGTTGTTTGCTTTTTGAATAACTTCTACCCAAAGGATATTAATGTTAAGTGGAAAATTGATGGATCAGAGAGGCAAAATGGTGTTTTGAATTCTTGGACTGATCAAGATTCTAAGGATTCAACATACTCTATGTCTTCAACTCTTACATTGACTAAGGATGAATACGAGAGACATAACTCTTACACATGTGAAGCTACTCATAAGACATCTACTTCACCTATCGTTAAATCTTTTAATAGGAATGAGTGCTCTGAGAAGGATGAATTG**TGA** |
| --- | --- |
| Heavy chain  (5'→3') | **ATG**GGTTGGTCATGTATCATCCTTTTTCTTGTTGCTACTGCTACAGGAGTTCATTCTGAACTTGAGTCAGGAGGTGGATTGGTTCAACCTGGTGGATCTCTTAAATTGTCATGCGCTGCTTCTGGTTTTACATTTTCTTCATACGGAATGTCTTGGGTTAGACAAACTCCTGATAAGAGGCTTGAATTGGTTGCTACAATTAATTCAAATGGTGGATCTACTTATTACCCAGATTCAGTTAAGGGAAGGTTTACTATCTCTAGGGATAACGCTAAGAACACTCTTTATTTGCAAATGTCTTCACTTAAGTCTGAGGATACTGCTATGTATTACTGTGCTAGAGAAGAGTATTACGGTAAAGCTTGGTTTGTTTATTGGGGTCAAGGAACACTTGTTACTGTTTCAGCTGCTAAGACTACACCACCTTCTGTTTACCCTTTGGCTCCAGGATCAGCTGCTCAAACAAATTCTATGGTTACTCTTGGTTGCTTGGTTAAAGGATATTTTCCTGAACCAGTTACTGTTACATGGAATTCAGGTTCTCTTTCTTCAGGAGTTCATACATTTCCTGCTGTTTTGCAATCAGATCTTTACACTTTGTCTTCATCTGTTACAGTTCCTTCATCTACTTGGCCATCTGAAACTGTTACATGTAATGTTGCTCATCCTGCTTCATCTACAAAGGTTGATAAGAAAATTGTTCCAAGGGATTGTGGATGCAAGCCTTGTATTTGCACTGTTCCAGAGGTTTCATCTGTTTTTATTTTTCCACCTAAGCCTAAGGATGTTCTTACTATCACATTGACTCCAAAGGTTACATGCGTTGTTGTTGATATCTCAAAGGATGATCCAGAAGTTCAATTTTCTTGGTTTGTTGATGATGTTGAGGTTCATACAGCTCAAACTCAACCTAGAGAAGAGCAATTCAATTCAACTTTTAGGTCAGTTTCTGAACTTCCAATTATGCATCAAGATTGGTTGAATGGTAAAGAGTTTAAATGTAGAGTTAACTCAGCTGCTTTCCCTGCTCCAATCGAAAAGACAATCTCTAAGACTAAAGGAAGGCCTAAAGCTCCACAAGTTTATACAATTCCACCTCCAAAAGAGCAAATGGCTAAGGATAAAGTTTCTCTTACATGCATGATCACTGATTTCTTTCCTGAAGATATCACTGTTGAGTGGCAGTGGAATGGTCAACCTGCTGAAAACTACAAGAACACACAACCAATTATGGATACTGATGGATCATACTTCGTTTACTCTAAGTTGAACGTTCAAAAGTCAAACTGGGAGGCTGGTAACACTTTTACTTGTTCTGTTCTTCATGAAGGATTGCATAATCATCATACTGAGAAGTCACTTTCTCATTCACCTGGAAAGTCTGAAAAAGATGAGTTG**TGA** |

The start and stop codons are shown in boldface letters, and the C-terminal codons encoding the endoplasmic reticulum-targeting signal SEKEDL are underlined.
